# Impaired Consciousness in a Coronavirus Disease 2019 Patient Caused by Low Serum Sodium: A Case Report

**DOI:** 10.31662/jmaj.2021-0023

**Published:** 2021-07-06

**Authors:** Kazuhiro Abe, Tomohito Nagai, Sayaka Ono, Masaru Komino, Kazuki Sato, Hikaru Nagahara, Kazuyuki Nishimura

**Affiliations:** 1 Department of Respiratory Medicine, Saiseikai Kurihashi Hospital, Kuki, Japan; 2 Department of Neurology, Saiseikai Kurihashi Hospital, Kuki, Japan; 3 Department of Gastroenterology, Saiseikai Kurihashi Hospital, Kuki, Japan

**Keywords:** COVID-19, SARS-CoV-2, neurological manifestation, PCR

## Abstract

A 30 year-old man with a high fever (37.5°C-40°C), vomiting, slurred speech, and mild cognitive impairment was admitted to our Emergency Department. He had traveled from Spain to the UK on business at the end of February 2020. A nasopharyngeal swab was positive by RT-PCR for severe acute respiratory syndrome coronavirus 2 (SARS-CoV-2), but a cerebrospinal fluid (CSF) sample was negative. His neurological abnormalities recovered completely on saline infusion to normalize his low serum sodium level. Although neurological abnormalities in patients with COVID-19 are rare, it is important to distinguish the etiologies including encephalitis, meningitis, or merely electrolyte abnormalities.

## Introduction

The majority of patients with COVID-19 manifest fever or body temperature > 37.5℃ and cough or shortness of breath, but neurological abnormalities have rarely been reported ^(1), (2), (3), (4), (5)^. Here, we report a 30 year-old man with SARS-CoV-2 infection with fever and disturbance of consciousness. He had slurred speech and mild cognitive impairment, with Glasgow Coma Scale and Japan Coma Scale (JCS) scores of 4 and I-1, respectively. His symptoms including neurological abnormalities recovered completely once his low serum sodium level was normalized by saline infusion, and he was discharged on the 13th hospital day.

We conclude that it is important to distinguish the etiology of neurological abnormalities in patients with COVID-19 because electrolyte imbalance may be inducing neurological manifestations.

## Case Report

A 30 year-old man was admitted to the hospital Emergency Department with high fever (37.5°C-40°C), vomiting, slurred speech, and mild cognitive impairment. He had been in Spain and the UK on business from February 23rd to March 1st, 2020 and experienced high fever and vomiting 3 days after returning to Japan. He was treated at a hospital on the fifth day after the symptom onset, but his symptoms persisted, and he was unable to retain any solids or liquids because of vomiting. His parents living in a distant part of the country noticed his slurred speech and called the emergency services on the seventh day after the onset of symptoms.

The patient was slightly confused on admission and showed mild cognitive impairment with slurred speech. He also had a brief convulsion episode on the first hospital day. Physical examination did not show any abnormalities of muscle tone, tendon reflex, and any pathological reflexes. We assessed his consciousness level using GCS14 or JCS I-1. His other vital signs, CBC, biochemical data, and arterial blood gas analysis are presented in Table 1.

**Table 1.** Laboratory Data on Admission.

**CBC**			**Biochemistry**			**BGA**		
WBC	5,900	/μL	Alb	3.4	g/dL	pH	7.572	
Neu	71.6	%	T-Bil	1.21	mg/dL	PCO_2_	37.7	mmHg
Lym	17.4	%	AST	171	U/L	PO_2_	80.7	mmHg
Mon	10.6	%	ALT	62	U/L	HCO_3_^-^	33.9	mEq/L
Eos	0.2	%	LDH	636	U/L	BE	11.0	mEq/L
Bas	0.2	%	γ-GTP	98	U/L	AG	14.8	mEq/L
RBC	602 × 10^4^	/μL	BUN	21	mg/dL			
Hb	18.3	g/dL	Cre	1.44	mg/dL			
Ht	49	%	Na	131	mEq/L			
Plt	190 × 10^3^	/μL	K	2.2	mEq/L			
			CRP	8.12	mg/dL			
**Nasopharyngeal Swab Test**					**CSF**			
Influenza type-A			(−)		Total Protein	43	mg/dL	
Influenza type-B			(−)		Albumin	27	mg/dL	
RS virus			(−)		Glucose	63	mg/dL	
Adenovirus			(−)		Color	clear		
SARS-Cov2 PCR			(+)		Nuclear cell count	< 1		
*S. pneumoniae(urine specimen)*			(−)		WBC	< 1		
*Legionella spp.(urine specimen)*			(−)		RBC	0		
					SARS-CoV2 PCR	(−)		

BGA, blood gas analysis; CSF, cerebrospinal fluid

**Figure 1.**
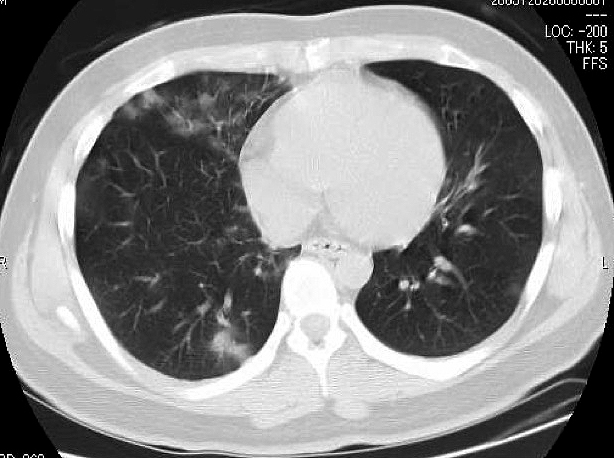
Chest CT on admission, indicating bilateral peripheral multiple ground glass infiltration.

A chest CT showed multiple areas of bilateral peripheral ground glass infiltration of the lungs (Figure 1). A brain MRI revealed no focal signs suggesting stroke and no encephalopathic signs such as encephalitis or meningitis. Taken together, we suspected COVID-19 and performed PCR for SARS-CoV-2 using a nasopharyngeal swab and a CSF sample. The swab tested positive, but the CSF was negative. The CSF data were as follows: clear appearance, 12.5 cmH_2_O initial pressure, nuclear cell count <1.0, 63 mg/dL glucose, and 43 mg/dL protein (Table 1).

**Figure 2.**
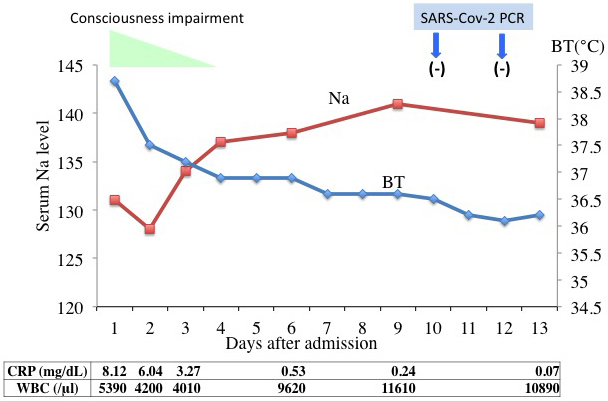
Clinical course. Consciousness impairment resolved as body temperature decreased and serum sodium level was normalized. Na, serum sodium concentration; BT, body temperature

From these data, we hypothesized that his neurological abnormalities were not directly caused by infection of the CNS by SARS-CoV-2 but by metabolic abnormalities such as a low serum sodium level. Accordingly, we corrected the sodium level by saline infusion, and by the fourth hospital day, his consciousness was almost completely normalized as his body temperature decreased and sodium level increased (141 mEq/L Na) (Figure 2). On the 13th hospital day, he was discharged after twice confirming SARS-CoV-2 negativity by PCR.

Written consent, including that for radiographic data, was obtained for publication.

## Discussion

Typical physical features of COVID-19 are general fatigue, fever and upper respiratory tract symptoms ^(1), (2), (3), (4), (5)^. Neurological abnormalities have rarely been reported, and only a young adult male with loss of consciousness caused by meningitis due to SARS-CoV-2 infection has been described in Japan ^(6)^.

Our patient experienced disturbances of consciousness but tested negative for SARS-CoV-2 PCR in a CSF sample and had no signs of encephalitis and meningitis by MRI. His neurological abnormalities resolved completely with correction of his serum sodium level. SARS-CoV-2 may induce neurological abnormalities by direct infection of the CNS or indirectly through metabolic abnormalities as demonstrated in this study or by other mechanisms such as immunological reactions. A recent study analyzing 58 consecutive patients with SARS-CoV-2 infection determined that 47 manifested encephalopathy, prominent agitation, confusion and corticospinal tract signs ^(7)^.

Another study in China analyzed 214 patients and identified 78 with neurological manifestations ^(8)^. In that study, impaired consciousness was significantly more common in more severe than in less severe disease (13 [14.8%] vs. 3 [2.4%]; P < .001).

Recently, ACE2 has been identified as a functional receptor for SARS-CoV-2. It is present in various organs including the CNS, peripheral nervous system and skeletal muscles ^(9), (10)^. Hence, some neurologic manifestations could be related to direct infection of the nervous system by SARS-CoV-2. In our case, there was no direct proof of CNS infection. Thus, it is likely that none of his neurological abnormalities were associated with CNS SARS-CoV-2 but were entirely due to the low serum sodium level caused by high fever, dehydration, and no food intake.

## Article Information

### Conflicts of Interest

None

### Author Contributions

KA: acquisition of data, analysis and interpretation of data, and drafting of the manuscript; TN, SO, MK, and KS: acquisition, analysis, and interpretation of data; and HN and KN: conception and design of the study, analysis and interpretation of data, and drafting or revision of the manuscript.
